# Fabrication, Characterization and Application of Biomolecule Micropatterns on Cyclic Olefin Polymer (COP) Surfaces with Adjustable Contrast

**DOI:** 10.3390/bios10010003

**Published:** 2019-12-28

**Authors:** Roland Hager, Thomas Haselgrübler, Sandra Haas, Anna-Maria Lipp, Julian Weghuber

**Affiliations:** 1 Center for Advanced Bioanalysis GmbH, Linz 4020, Austria; thomas.haselgruebler@cbl.at (T.H.); Sandra.Haas@profactor.at (S.H.); annama.lipp@gmail.com (A.-M.L.); 2 University of Applied Sciences Upper Austria, Stelzhamerstrasse 23, Wels A-4600, Austria

**Keywords:** micropatterns, photolithography, protein–protein interaction, micropatterned polymers, cyclic olefin polymer, total internal reflection fluorescence (TIRF)

## Abstract

Peptide and protein micropatterns are powerful tools for the investigation of various cellular processes, including protein–protein interactions (PPIs). Within recent years, various approaches for the production of functional surfaces have been developed. Most of these systems use glass as a substrate, which has several drawbacks, including high fragility and costs, especially if implemented for fluorescence microscopy. In addition, conventional fabrication technologies such as microcontact printing (µCP) are frequently used for the transfer of biomolecules to the glass surface. In this case, it is challenging to adjust the biomolecule density. Here, we show that cyclic olefin polymer (COP) foils, with their encouraging properties, including the ease of manufacturing, chemical resistance, biocompatibility, low water absorption, and optical clarity, are a promising alternative to glass substrates for the fabrication of micropatterns. Using a photolithography-based approach, we generated streptavidin/biotinylated antibody patterns on COPs with the possibility of adjusting the pattern contrast by varying plasma activation parameters. Our experimental setup was finally successfully implemented for the analysis of PPIs in the membranes of live cells via total internal reflection fluorescence (TIRF) microscopy.

## 1. Introduction

Micropatterned protein or DNA substrates have been successfully used to address many different biological questions in various research areas, including disease diagnosis, clinical and pharmacogenomics research, the analysis of cellular functions, and drug discovery [1,2,3,4]. In this regard, micropatterned surfaces as part of lab-on-a-chip systems are of special interest as they can fulfill the rising demand for high-throughput diagnostic tools. In microfluidic systems, small volumes of reagents and samples are moved through channels and reactors miniaturized in a chip. Various assays and procedures have already been embedded in these systems, such as immunoassays, enzymatic assays, polymerase chain reaction (PCR), DNA sequencing, cell counting, and cell sorting [5,6,7]. Importantly, the increasing interest in microfluidic devices boosts the development of lab-on-a-chip systems based on thin and elastic foils (lab-on-a-foil system) as an alternative to glass surfaces [8]. After specifying the intended application of a microfluidic system, the choice for a certain fabrication method and material has to be made. Properties such as Young’s modulus, tensile strength, chemical resistance, biocompatibility, water adsorption, gas permeability, autofluorescence, and light transmission play an important role in the choice of the proper material. Due to its high elasticity, suitability for mass production, low costs, and good optical properties, including applicability for total internal reflection fluorescence microscopy (TIRF) and thermoplastic materials such as cyclic olefin polymers (COPs) are promising alternatives to glass surfaces.

For the generation of functional protein and DNA micropatterned surfaces, different approaches are used. A popular soft lithographic technique is microcontact printing (µCP). Here, the elastomeric material poly (dimethylsiloxane) (PDMS) is used as a patterned stamp coated with biomolecules that are transferred to a reactive or adhesive substrate surface with face-to-face contact [9,10]. Patterning resolution in the nanometer range can be realized with the µCP approach [9]. This approach is robust, easy to implement, inexpensive, and well established. However, there are several limitations of the µCP approach, such as the restriction in the aspect ratio of the stamp features due to the deformability of PDMS. This can lead to a mechanical collapse or the sagging of the stamp structures during the printing process that result in irregular shapes and patterns [11]. Another disadvantage negatively influencing the performance of PDMS is the shrinkage of approximately 1% upon curing and swelling by solvents such as hexane, diethyl ether, and toluene [12,13]. These limitations can preclude the reproducible formation of submicron features [14]. To solve these problems, alternative patterning techniques, such as dip-pen nanolithography (DPN), polymer pen lithography (PPL), photolithography, and nanoimprint lithography (NIL), have been successfully developed [15,16,17,18].

As mentioned before, micropatterns are mostly created on glass or silicon surfaces, but COPs represent a versatile alternative to these conventional materials, allowing for the mass fabrication of microfluidic systems at low costs [19]. The performance of biological assays strongly depends on the substrate material. This can influence DNA and protein adhesion, growth, and cell behavior. Numerous types of biological samples have been positively used for analysis on COP substrates [19]. COP surfaces that have been modified by plasma activation achieved comparable performance in cell growth assays to commercial tissue culture polymers [20,21]. It has been shown that polymer surfaces treated with plasma serve as a suitable substrate for binding and patterning biomolecules [22,23]. Taken together, based on their beneficial properties, these findings show that COPs have an emerging role in the fabrication of microfluidic systems [24], especially in terms of surface functionalization and biomolecule immobilization [25,26,27].

Here, we describe the application of a photolithographic method for the generation of micropatterns on COP substrates. By varying the plasma treatment time, we successfully used this system for the formation of biomolecule patterns with high contrast. We demonstrate the applicability for the quantitation of PPIs in live cells with total internal reflection fluorescence (TIRF) microscopy, as previously reported [28,29,30,31].

## 2. Materials and Methods

### 2.1. Chemicals

All chemicals were from Sigma-Aldrich Handels GmbH (Vienna, Austria) unless noted otherwise. Cyclic olefin polymer (COP; Zeonor, Tokyo, Japan) foils with a thickness of 188 µm were obtained from microfluidic ChipShop GmbH (Jena, Germany). Positive photoresist G2 S1818 and developer ma-D 331S were obtained from micro resist technology GmbH (Berlin, Germany). Bovine serum albumin (BSA) was obtained from SERVA Electrophoresis GmbH (Heidelberg, Germany). Streptavidin-Cy5 and biotinylated epidermal growth factor (EGF) were purchased from Life Technologies (Vienna, Austria). EGFR-GFP was a kind gift from Alexander Sorking (Addgene 32751) [20]. FLAG peptide (DYDDDK) with N-terminal biotin and 4,7,10-trioxa-1,13-tridecanediamino succinic acid (Ttds) linker was synthesized by JPT Peptide Technologies (Berlin, Germany). Anti-FLAG antibody was obtained from Agilent (Vienna, Austria) and labeled using an Alexa Fluor 488 antibody labeling kit (Life Technologies). Goat anti-mouse IgG:FITC was obtained from AbD Serotec (Bath, UK), and biotin mouse anti-human CD71 was obtained from BD Biosciences (San Jose, CA, USA). RPMI 1640 medium, fetal bovine serum (FBS), L-glutamine, Genectin^TM^ Selective Antibiotic (G418), penicillin, and streptomycin were obtained from ThermoFisher Scientific (Waltham, MA, USA). Milli-Q water (18.2 MΩ) was used throughout. 

### 2.2. Preparation of Micropatterned Peptide Surfaces

**Figure A1:**
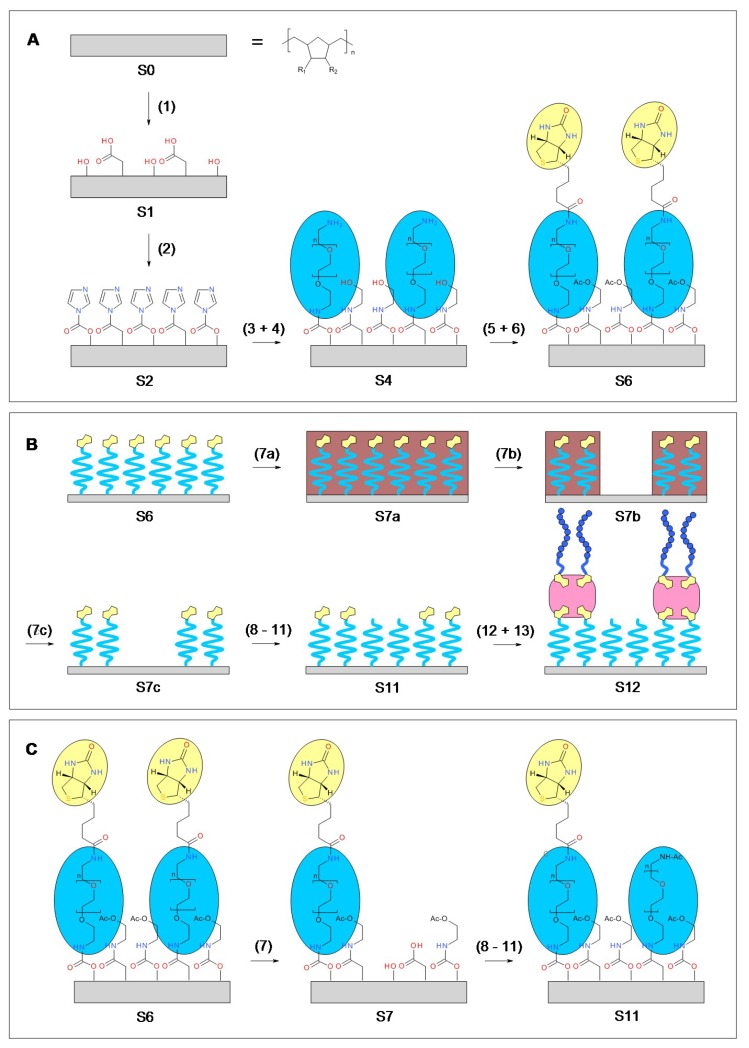
Schematic overview of surface chemistry and micro-patterning procedure on COP substrate. (**A**) Fabrication of PEG layer with terminal biotin groups on COP surface. (**B**) Micro-patterning process via photolithography leading to a micropatterned biotin-PEG layer. (**C**) Surface chemistry before and after micro-patterning.

A detailed procedure for the surface preparation of micropatterned COPs is described in the Appendix A and shown in Figure A1. Briefly, COP foils were washed with ethanol and ddH_2_O before hydrophilization by plasma oxidation. Subsequently, hydrophilized COP foils were activated with 1,1’-carbonyldiimidazole (CDI) before grafting with poly(ethylene glycol) diamine (PEG diamine) by incubation with Jeffamine ED 600. Residual CDI-activated groups on the PEG-grafted foils were blocked with ethanolamine followed by the biotinylation of terminal amine groups on the tethered PEG chains with biotin NHS-ester. Finally, residual free amine and hydroxyl groups were blocked by acetylation.

Positive photoresist G2 S1818 was used to generate micropatterns via photolithography. Photoresist was spin-coated onto PEG-grafted COP foil to a layer thickness of 2 µm according to the manufacturer’s protocol. After prebaking the photoresist, the slides were exposed at 365 nm using an EVG 620 mask alignment system followed by photoresist development. The developed slides were plasma-etched at 20 W or 30 W for 1 min to 20 min. By varying the plasma power and plasma-etching time, the binding capacity of the photoresist-embedded biotinylated PEG layer could be adjusted.

Photoresist was removed by washing in acetone, ethanol, and ddH_2_O. Subsequently developed areas of micropatterned COP foils were activated with CDI, grafted with PEG, blocked with ethanolamine, and acetic anhydride as previously described. This was followed by another blocking step with 1% bovine serum albumin (BSA) before incubation with streptavidin and Cy5-labeled streptavidin. Finally, biotinylated biomolecules such as biotinylated c-myc, biotinylated mouse anti-human CD71, and biotinylated FLAG peptide diluted in phosphate buffered saline containing 0.05% Tween-20 (PBST) were pipetted onto the surfaces. For the detection of the biotinylated antibodies, the corresponding fluorescently labeled antibodies were used, such as monoclonal anti-c-myc, goat anti-mouse IgG:FITC, or anti-FLAG antibody.

### 2.3. Cell Culture and Transfection

Jurkat cells (Jurkat E6.1 TIB-152, ATCC, Manassas, VA, USA) were maintained in complete RPMI 1640 medium supplemented with 10% FBS, 2 mM L-glutamine, 100 units/mL penicillin, and 100 mg/mL streptomycin at 37 °C and 5% CO_2_. Cells were transfected with 2 µg of plasmid DNA by nucleofection (Lonza, Cologne, Germany). GFP-positive cells were enriched by flow cytometry using a FACS Aria^TM^ cell sorter (BD Biosciences, San Jose, CA, USA), and positive Jurkat cells were selected in complete medium with 400 µg/mL G418.

### 2.4. Fluorescence Microscopy and Assay Readout

Fluorescence microscopy was performed using an Axiovert 200 microscope equipped with a mercury lamp HBO100 (both from Zeiss, Jena, Germany) and appropriate filter sets (AHF Analysentechnik, Tübingen, Germany). Fluorescence emission was collected via a 40× Neofluar objective (Zeiss) and detected using a charge-coupled device (CCD) camera (Photometrics, Tucson, AZ, USA).

Live-cell experiments were performed in serum-free HBSS at 37 °C adjusted by an objective heating system (PeCon, Erbach, Germany) using a 100× α-Plan-Apochromat objective (NA = 1.46, Zeiss) on a modified Axiovert 200 microscope (Zeiss). For TIRF microscopy, the fluorescence of GFP or Cy5 was excited by a Kr^+^/Ar^+^ mixed gas laser (Innova, Coherent, Santa Clara, CA, USA) at 488 nm or 647 nm, respectively. Samples were illuminated in a TIRF configuration using a TIRF condenser (Till-Photonics, Gräfelfing, Germany) and a custom superflat beamsplitter (BS-zt488/647/780rpc). Emitted fluorescence was split into 2 channels using a beamsplitter (HC BS580), filtered using HC525/45 and ET700/75 bandpass filters, and imaged simultaneously on two CCD cameras (CoolSnap HQ, Photometrics, Tucson, AZ, USA). All filters were obtained from AHF Analysentechnik (Tübingen, Germany).

### 2.5. Data Analysis

Image processing and analysis were performed using ImageJ (NIH, Bethesda, MD, USA) and Microsoft Office Excel 365 (Redmond, Washington D.C., USA). The results were expressed as the mean ± SD. The contrast was calculated using the following formula: contrast = (F_max_ − F_min_)/(F_max_ − BG) [30]. F_max_ and F_min_ represent the fluorescent counts in the bright grid areas (on regions) and the dark, dot areas (off regions), respectively. BG is the background, which refers to a micropatterned COP foil not incubated with fluorescent molecules.

## 3. Results and Discussion

### 3.1. Fabrication of Micropatterned COP Foils Using Photolithography Followed by the Immobilization of Biomolecules

For lab-on-a-chip applications, silicon and glass are the first materials used. The fabrication technology for these materials is well established because it was adopted from the microelectronics industry [32]. The major drawbacks of functionalized glass include high costs and especially its fragility, as some fluorescence microscopy techniques, including TIRF, call for glass coverslips (~180 µm thickness) [33]. Here, we describe an alternative material [34], cyclic olefin polymer (COP), for the fabrication of micropatterned substrates based on a photolithographic approach. A disadvantage of the application of COP foils in combination with TIRF microscopy is its low rigidity, which is crucial for this microscopy technique. Therefore, COP foils have to be stabilized by the application of polymer castings as used for microwell plates or press-to-seal silicone isolators as described in the Appendix A [8,35]. With surface stabilization, focal drift caused by polymer flexibility can be eliminated.

Figure A1 indicates the generation of micropatterned COP surfaces. Based on a previous study describing a surface fabrication process on glass slides [16], the starting point for our protein- and peptide-based micropatterned polymer substrate was a 188 µm thick, air-plasma-activated COP foil. Air plasma treatment generated a high density of oxygen-containing functional groups, such as carboxyl and hydroxyl groups [36,37]. These functional groups were employed to form a dense and homogeneous PEG layer on the COP surface using PEG diamine and the activation of the surface-bound carboxyl and hydroxyl groups with CDI. The PEG layer was required to prevent the nonspecific adhesion of peptides and proteins. Terminal amine groups of the PEG chains were functionalized with biotin and used for the immobilization of biotinylated biomolecules via the biotin–streptavidin interaction.

The biotin-functionalized PEG layer was embedded within a thin film of positive photoresist, which was exposed to UV light via a photomask. After the removal of the UV-exposed photoresist with developer, the biotinylated PEG layer in the photoresist-free dot areas was eliminated by plasma treatment. As a major advantage, our experimental setup allows for a contrast adjustment of the patterned molecules by adapting the plasma settings during the fabrication process: increasing the plasma treatment time leads to a better formation of a dense PEG layer and thereby reduced unspecific binding of biomolecules. The respective critical step is described in detail in Figure A1, Appendix A. A maximum contrast appears favorable if the surface is used for the quantitation of weak/transient PPIs in live cells, such as receptor tyrosine kinases and their intracellular binding partners [28,38]. A reduced density of the PEG layer might be preferable if particular molecules such as fibronectin, intercellular adhesion molecules (ICAMs), or laminin, which improve cell adhesion, also need to be present in the dot area [39]. Sufficient cell adhesion in both the grid and the dot regions is a prerequisite for TIRF-based PPI quantitation, as inhomogeneous adhesion—dependent on the cell type—leads to insufficient excitation in the evanescent field and thereby false-positive results [40].

**Figure 1:**
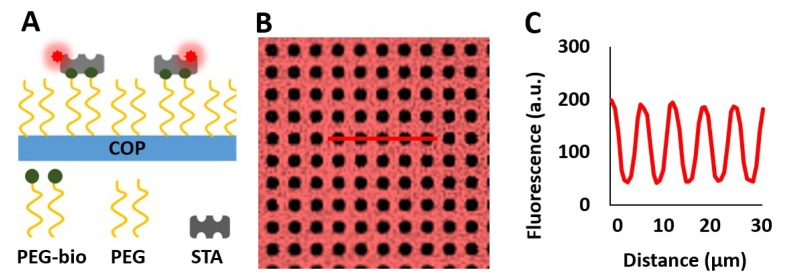
Determination of pattern contrast and biomolecule density on micropatterned substrates using fluorescence microscopy. (**A**) Schematic overview of fluorescently labeled streptavidin bound to the micropatterned cyclic olefin polymer (COP) surface. (**B**) Fluorescence image (image size: 65 × 65 µm) and (**C**) the corresponding line profile.

**Figure A2:**
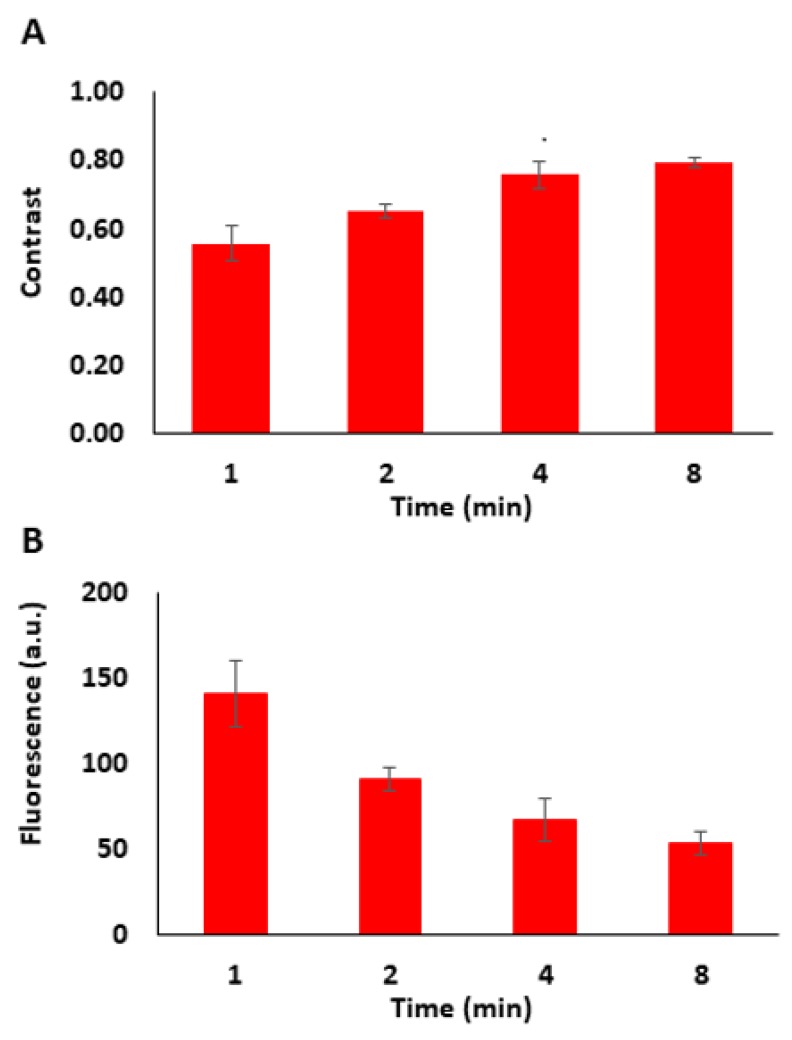
Determination of pattern contrast (**A**) and fluorescence intensity (**B**) of micropatterned fluorescently labeled streptavidin on COP foils via fluorescence microscopy depending on plasma treatment time.

The fluorescence intensity served as a measure of streptavidin, peptide, or protein density on the micropatterned COP surface. A schematic representation of fluorescently labeled streptavidin bound to the micropatterned biotin-PEG layer is shown in Figure 1A. We measured streptavidin pattern contrast using Cy-5-labeled streptavidin (see Figure A2). The contrast between the streptavidin-bound and nonbound areas was increased by up to 0.79 ± 0.03 for the 8 min plasma treatment time. A fluorescence microscopy image of a representative micropatterned surface labeled with streptavidin-Cy5 molecules is shown in Figure 1B. Figure 1C demonstrates the corresponding line profile of the highly specific binding of fluorescently labeled streptavidin-Cy5 molecules on the COP surface.

**Figure 2:**
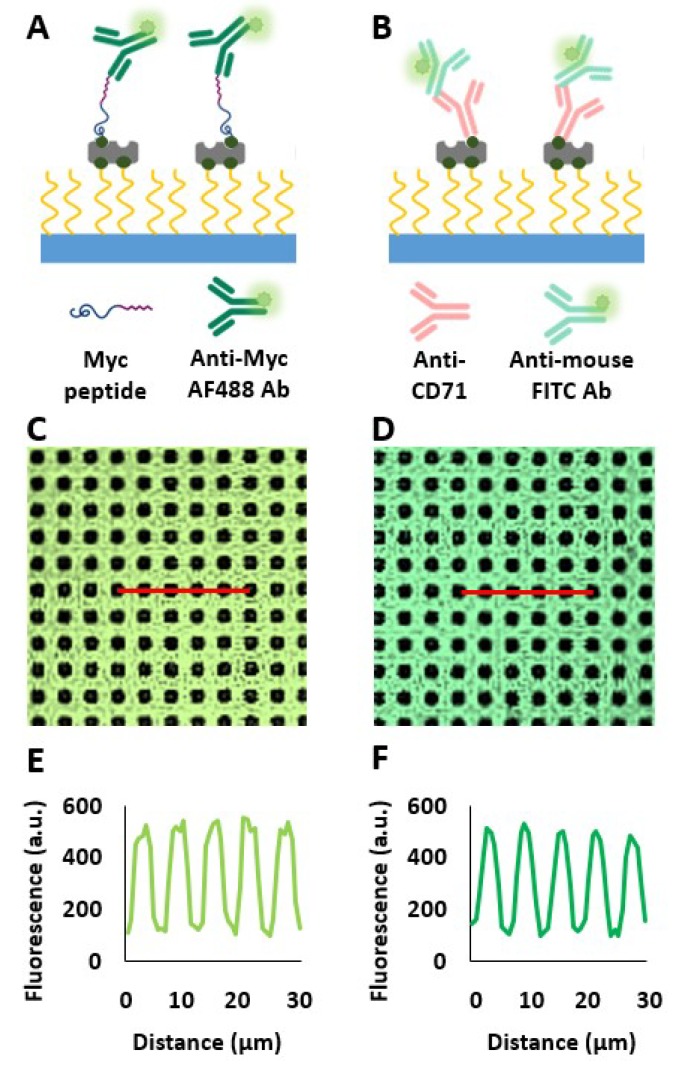
Determination of pattern contrast and biomolecule density on micropatterned substrates using fluorescence microscopy. (**A**) Schematic overview of micropatterned Myc peptide using anti-Myc AF488 Ab for detection. (**C**) Fluorescence image (image size: 65 × 65 μm) and (**E**) the corresponding line profile. (**B**) Schematic overview of micropatterned anti-CD71 using anti-mouse FITC Ab for detection. (**D**) Fluorescence image (image size: 65 × 65 μm) and (**F**) the corresponding line profile.

**Figure A3:**
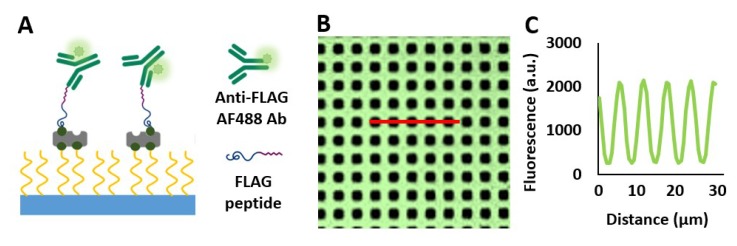
Determination of pattern contrast and biomolecule density on micropatterned substrates using fluorescence microscopy. (**A**) Schematic overview of anti-FLAG-AF488 binding to micropatterned FLAG peptide. (**B**) Fluorescence image (image size: 65 × 65 µm) and (**C**) corresponding line profile.

Peptide and protein micropatterns were further characterized by incubating the streptavidin micropatterned COP foil with Myc peptide and Anti-CD71, respectively. For the detection of the immobilized biomolecules, anti-Myc AF488 or anti-mouse FITC antibodies were used. A schematic overview of the micropatterned peptide and protein surface is shown in Figure 2A,B. Microscopy images and corresponding line profiles are shown in Figure 2C–F. In Figure A3, the immobilization of FLAG peptide onto the micropatterned surface is indicated. We used anti-FLAG AF 488 as a detection antibody for the determination of the fluorescence contrast. Peptides and proteins were bound to the grid area at high density. Unspecific binding to the PEG-grafted dot areas was very low, resulting in a high pattern contrast. The low fluorescence levels in the dot areas indicate that unspecific peptide or protein binding can be reduced to a very low level. This results in a very high pattern contrast suitable for the quantitation of weak/transient PPIs.

### 3.2. Tuning the Fluorescence Intensity of Immobilized Biomolecules

**Figure 3:**
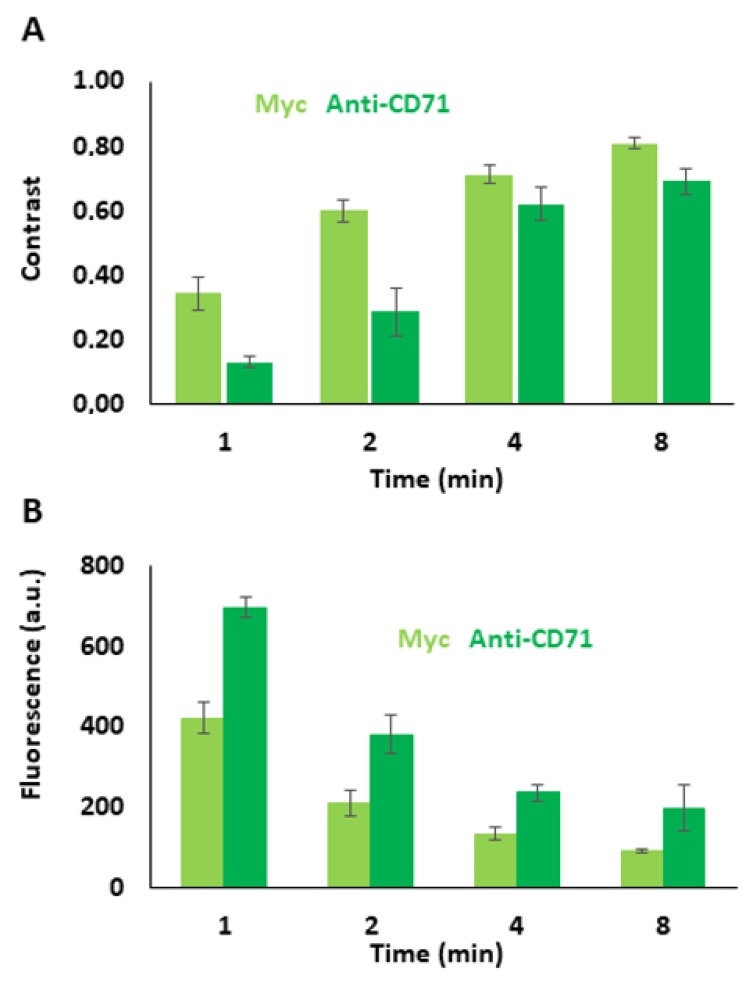
(**A**) Correlation between plasma treatment time and contrast using different labeled fluorescence biomolecules. (**B**) Correlation between plasma treatment time and fluorescence intensity of the dot areas.

As mentioned before, PEG layer formation is critical for the applicability of the micropatterned surface [41,42]. Therefore, we determined the influence of plasma treatment parameters on surface functionality. The percentage of removal of biotinylated PEG molecules in the photoresist-free dot areas is a function of the plasma treatment. We increased plasma treatment time at an intensity of 20 W to decrease specific and nonspecific binding in the dot areas, thereby increasing the micropattern contrast. Different contrast levels for Myc peptide as well as for anti-CD71 are shown in Figure 3 and for fluorescently labeled streptavidin in Figure A1. The contrast between bound and nonbound anti-Myc AF488 can be adjusted between 0.34 ± 0.11 for 1 min plasma treatment time up to 0.81 ± 0.03 for 8 min plasma treatment time, respectively. For anti-mouse FITC, Ab contrast values range from 0.13 ± 0.03 for 1 min to 0.69 ± 0.08 for 8 min plasma treatment time.

**Figure A4:**
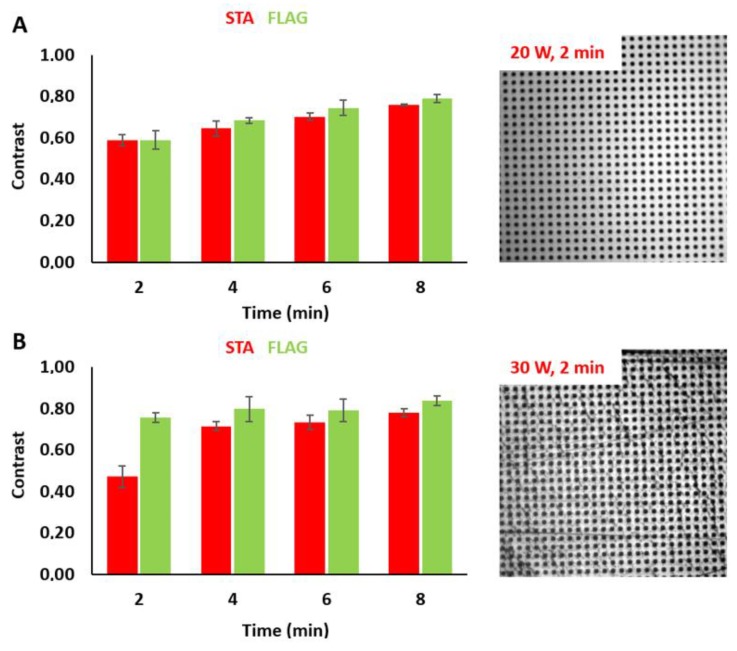
Correlation between plasma treatment time and contrast using different plasma intensities. (**A**) 20 W and (**B**) 30 W. Representative images were obtained at both intensities after 2 min of plasma treatment.

**Figure A5:**
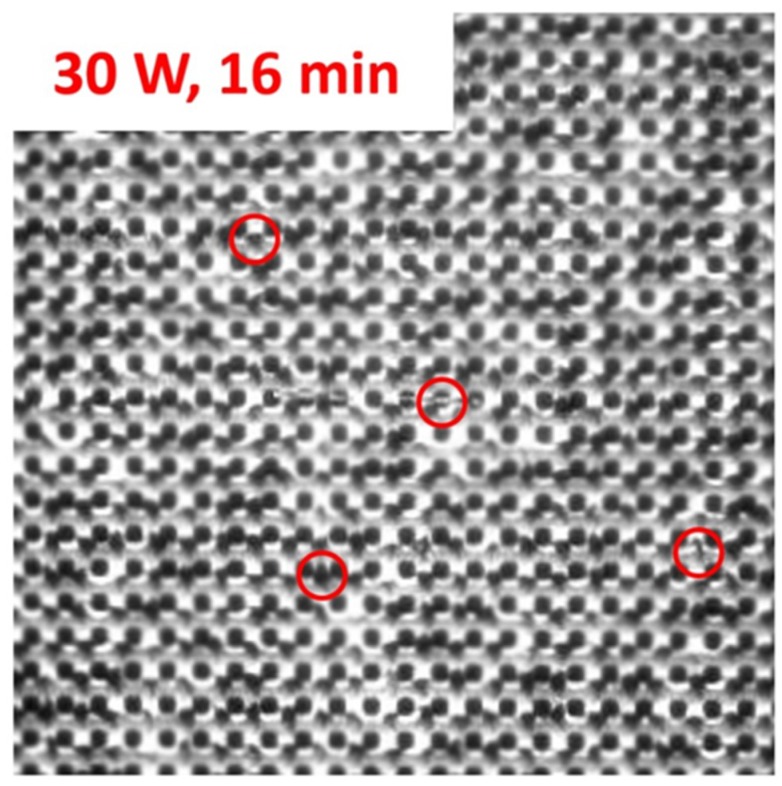
Representative image obtained after long-term plasma treatment (16 min, 30 W). Red circles indicate examples of irregular pattern formation.

Fluorescence intensity in the dot areas can also be decreased by increasing plasma intensity [43,44]. Nonspecific binding was reduced to a minimum in the dot areas of the micropatterns, whereas the fluorescence intensity in the grid area remained stable. In Figure A4, different plasma intensities (20 W and 30 W) are compared. As shown in the two representative images, the adjustment of the contrast at a plasma intensity of 20 W provided the best results. Application of 30 W resulted in an increased number of pattern defects and adverse pattern homogeneity. When plasma intensity was too high or plasma treatment time was too long, micropatterns appeared blurred and diffuse. Figure A5 shows a representative image obtained after long-term plasma treatment (16 min, 30 W) indicating irregular pattern formation.

### 3.3. Specific Receptor Interactions in a Live-Cell Context

**Figure 4:**
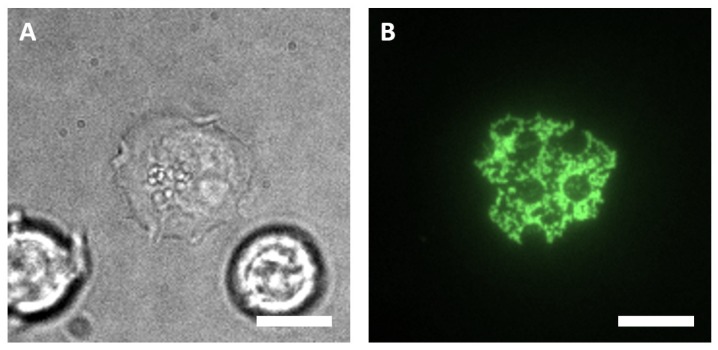
Testing for specific protein–receptor interaction. Epidermal growth factor (EGF) is immobilized onto the whole surface except on the PEGylated dot areas. Bright-field image (**A**) and representative TIRF image (**B**) of a Jurkat cell expressing EGFR-GFP on the EGF micropatterned COP surface. Scale bar: 10 µm.

To provide proof of principle for live-cell assays on our generated COP substrates, we tested the micropatterned surface for the detection of the protein–receptor interaction of immobilized biotinylated proteins (see Figure 4). The successful redistribution of a receptor by specific interaction with patterned ligands or antibodies represents the core principle of the so-called ‘micro-patterning’ assay that has been developed and further improved in our lab within the last years, enabling the quantitation of protein–protein interactions [28,40]. Therefore, we studied the interaction of epidermal growth factor (EGF) with its receptor (EGFR) in the human leukemic suspension cell line Jurkat [16]. Cells expressing EGFR-GFP were grown on immobilized biotinylated EGF micropatterns. As shown in Figure 4, the cells attached sufficiently to the surface, and EGFR-GFP was found mainly in the EGF immobilized area. This result confirms the suitability of the micropatterned COP surface for the TIRF-based analysis of PPIs.

## 4. Conclusions

In summary, we developed micropatterned surfaces on COP substrates with adjustable biomolecule pattern contrast. The functionalization with bioactive proteins allows, for example, the validation of receptor interactions in their natural environment. As proof of principle, we tested the interaction of EGF with EGFR on the micropatterned COP substrate in a live-cell assay. By changing the plasma settings (plasma energy and treatment time) in the fabrication process, we demonstrated how this influences the reactivity of the surface. We present conditions that reduce unspecific binding in dot areas by PEGylation-based passivation, which results in high contrast of the desired streptavidin grids. Finally, we demonstrate that PPIs in living cells can also be evaluated by TIRF microscopy on micropatterned COP substrates produced by a photolithographic approach. 

## Appendix A

**Fabrication of Micropatterned Biomolecule Patterns on COPs**

The number in brackets for each reaction step, e.g., poly(ethylene glycol) (PEG)-grafting (1), refers to the respective reaction arrows in Figure A1. S0, S1, etc. relate to the different surfaces prepared in the individual steps (S1 = surface after reaction step 1, etc.). All reactions involving fluorophores were performed in the dark.

**Plasma Oxidation of COP Foil (1)**

COP foils (S0) washed sequentially in acetonitrile (2×), ethanol (2×), and water (2×) were spin-dried by centrifugation and hydrophilized by plasma oxidization at 100 W for 120 s (S1).

**Activation with 1,1’-Carbonyldiimidazole (2)**

Hydrophilized COP foils (S1) were activated with 1,1’-carbonyldiimidazole (CDI) (5% in diglyme = 50 mg/1 mL diglyme for 10 foils). Approximately 100 µL of reaction solution was pipetted onto each foil arranged in stacks of up to 10. The foils were placed in a desiccator containing silica gel and allowed to react at room temperature (RT) in the dark for 6 h. The foils were washed sequentially in diglyme, ethanol, and finally spin-dried (S2).

**COP Foils PEG-Grafting (3)**

CDI-activated COP foils (S2) were PEG-grafted by incubation with Jeffamine ED-600. A total of 150 µL of Jeffamine ED-600 was pipetted onto each of the foils assembled in stacks of up to 10. The foils were allowed to react at room temperature (RT) in the dark for 24 h. Finally, the foils were washed in 1% aqueous acetic acid (2×, 5 min) and H_2_O (2×, 1 min), with sonication once in each washing solution for 5 min.

**Blocking with Ethanolamine (4)**

Residual CDI-activated groups on PEG-grafted foils (S3) were blocked by incubating the foils in 50 mM ethanolamine in 0.1 M TRIS buffer (pH 9) using a Teflon reactor with a removable slide rack and stirring option. After a reaction time of 1 h, the foils were washed in H_2_O (3×, 5 min) and spin-dried.

**Biotinylation (5)**

Blocked PEG-grafted foils bearing terminal amine groups (S2) were reacted with biotin NHS-ester (10 mg) dissolved in a mixture of dimethyl sulfoxide (DMSO, 100 mL) and *N*,*N*-diisopropylethylamine (DIPEA, 3 mL) at RT overnight. Excess biotin reagent was removed by washing the slides sequentially in DMSO (3×, 5 min), borate buffer (pH 8.4, 1×, 30 min with stirring 1×, 5 min), and H_2_O (2×, 5 min). Finally, the foils were spin-dried.

**Blocking with Acetic Anhydride (6)**

Free amine and hydroxyl groups were blocked by acetylation using acetic anhydride (10 mL), DIPEA (2 mL), and 4-(dimethylamino)pyridine (DMAP) (30 mg) dissolved in acetonitrile (MeCN, 90 mL). After reaction at RT for 4 h, the slides were washed sequentially in MeCN, EtOH, and H_2_O, and finally spin-dried (S4).

**Micro-Patterning with Positive Photoresist (7)**

A 2 µm thick layer of photoresist G2 S1818 was spin-coated (2000 rpm, 60 s) onto the PEG-grafted foil bearing terminal biotin groups (S5a) according to the manufacturer’s protocol. After pre-baking at 115 °C for 1 min, the slides were exposed at 365 nm (15 mW/cm^2^) for 4 s using an EVG 620 mask aligner (EV Group, Schärding, Austria) through a chromium photomask Photronics MZD GmbH (Dresden, Germany) with 3 µm squares and 3 µm spacing. The photoresist was developed for 60 s using developer ma-d331s (S5b). To check the quality of the photoresist features, each slide was quality-checked using light microscopy. Slides that showed over- or underdeveloped micropatterns were discarded. Subsequently, developed slides were plasma-etched at different energy between 1 min and 20 min using a NANO plasma cleaner (type Nano, Version B, low-pressure plasma system equipped with a 40 kHz generator, power: 0–1000 W, Diener electronic GmbH, Ebhausen, Germany). The plasma process was performed at room temperature. Photoresist was then removed by sequential washes in acetone and ethanol. Finally, the slides were rinsed with H_2_O and spin-dried (S5c). Surface characterization was performed by atomic force microscopy as described previously [15] to rule out large height differences between PEGylated and non-PEGylated areas that would negatively influence the readout in TIRF configuration.

**Activation of the Developed Areas with 1,1’-Carbonyldiimidazole (8)**

Micropatterned COP foils (S7c) were activated in the developed areas using the protocol for CDI activation described in Section 2.2.

**PEG-Grafting in the Developed Areas (9)**

Foils CDI-activated in the developed area (S8) were PEG-grafted using the protocol detailed in Section 2.3.

**Blocking with Ethanolamine (10)**

Residual CDI-activated groups in the developed area (S8) were blocked with ethanolamine using the protocol described in Section 2.4.

**Blocking with Acetic Anhydride (11)**

Amine groups in the developed area (S8) were blocked with acetic anhydride using the protocol detailed in Section 2.5.

**Streptavidin (STA) Conjugation (12)**

Micropatterned biotin foils (S6) were blocked with 1% BSA in 10 mL PBST (PBS containing 0.05% Tween-20) for 30 min. The foils were washed in PBST, H_2_O and spin-dried before incubation with a mixture of STA and Cy5-labeled STA solution (100 µg/mL STA + 10 µg/mL STA-Cy5 in PBST) at RT for 1 h. The reaction was performed by assembling two foils with their micropatterned sides face-to-face using 200 µL of STA/Cy5-labeled STA solution. After washing in PBST and H_2_O, the foils were spin-dried and stored in a petri dish filled with argon and sealed with Parafilm at 4 °C until use.

**Biomolecule Immobilization (13)**

Press-to-seal silicone isolators (Sigma-Aldrich, 12 chambers, 2 mm diameter, 2 mm height) were mounted on micropatterned STA foils (S10). Biotinylated biomolecules diluted in PBST (20 µL, 100 µg/mL) were pipetted into chambers of interest. After incubation at RT for 1 h, the chambers were washed with 100 µL PBST and 100 µL PBS.
